# Reducing Osteopenia of Prematurity-related Fractures in a Level IV NICU: A Quality Improvement Initiative

**DOI:** 10.1097/pq9.0000000000000723

**Published:** 2024-04-03

**Authors:** Linsey Cromwell, Katherine Breznak, Megan Young, Anoosha Kasangottu, Sharon Leonardo, Catherine Markel, Andreea Marinescu, Folasade Kehinde, Vilmaris Quinones Cardona

**Affiliations:** From the * Department of Pediatrics St Christopher’s Hospital for Children, Philadelphia Pa.; † Department of Clinical Nutrition, St Christopher’s Hospital for Children, Philadelphia Pa.; ‡ Department of Pharmacy, St Christopher’s Hospital for Children, Philadelphia Pa.; § Department of Pediatrics, Drexel University College of Medicine, Philadelphia Pa.; ¶ Department of Nursing, St Christopher’s Hospital for Children, Philadelphia Pa.

## Abstract

**Background:**

Osteopenia of prematurity (OOP) is often a silent disease in the neonatal intensive care unit (NICU). Despite its association with increased neonatal morbidity, such as fractures, wide variation exists in screening, diagnostic, and management practices. We sought to decrease the rate of OOP-related fractures in our level IV NICU by 20% within 1 year.

**Methods:**

A multidisciplinary quality improvement team identified inconsistent screening, diagnosis, and management of OOP, as well as handling of at-risk patients, as primary drivers for OOP-related fractures. Using the model for improvement, we implemented sequential interventions, including screening, diagnosis, and a management algorithm as a “handle-with-care” bundle in infants at risk for fractures.

**Results:**

194 at-risk infants were included, 59 of whom had OOP. There was special cause variation in OOP-related fractures, with a reduction from 0.43 per 1000 patient days to 0.06 per 1000 patient days with our interventions. There was also an improvement in days between fractures from 62 to 337 days. We achieved these improvements despite a similar prevalence of OOP throughout the initiative. We showed special cause variation with increased patients between missed OOP documentation and improved collection of OOP screening laboratories at 4 weeks of life without increased blood testing.

**Conclusion:**

A multidisciplinary team approach with standardized OOP screening, diagnosis, and management guidelines, including a handle-with-care bundle, reduces OOP-related fractures in a level IV NICU.

## INTRODUCTION

Osteopenia of prematurity (OOP), also known as metabolic bone disease of prematurity (MBDP), is a prevalent but silent disease in the neonatal intensive care unit (NICU). OOP is defined as reduced bone mineralization leading to poor skeletal support and is estimated to occur in up to 23% of very low birthweight and 55% of extremely low birthweight infants.^1^ Infants born prematurely are at risk for OOP due to lack of calcium and phosphorous accretion, which most commonly occurs during the third trimester.^2^

Antenatal and postnatal risk factors are involved in the development of OOP. Antenatal factors include placental insufficiency, preeclampsia, and chorioamnionitis, while postnatally bronchopulmonary dysplasia, necrotizing enterocolitis, prolonged parenteral nutrition (PN), and medications such as diuretics, methylxanthines and glucocorticoids prevail.^2^

Biochemical markers used to screen for bone density loss accurately depict bone demineralization around 4–6 weeks of life.^1^ Alkaline phosphatase (ALP) is a known marker of bone turnover; however, it is often also elevated in liver dysfunction and particular gastrointestinal (GI) pathologies, and there is variation in its proposed diagnostic cutoff value. OOP screening guidelines vary widely, especially involving diagnostic thresholds for biochemical markers and imaging modalities to measure bone density.^3,4^ Lack of consensus on screening and management strategies makes standardization of care challenging. OOP is associated with increased ventilator dependence and fractures in the short term, as well as abnormal bone development and poor growth in the long term.^5–7^ Such morbidity can indirectly implicate OOP in increasing healthcare costs.

In our referral level IV NICU, we noted multiple reports of patients with fractures between January 2019 and January 2020. A focused review of each case revealed all patients had a unifying diagnosis of OOP. In addition, 2 patients had a remote history of central line placement in the same extremity as the fracture. Given OOP was a commonality among all cases, further review of all infants at risk for OOP revealed variation in OOP screening practice and inconsistency in diagnosis. With this in mind, we aimed to decrease the rate of fractures by standardizing OOP screening and management practices in our level IV NICU by 20% within 1 year of project initiation.

## METHODS

### Context

St. Christopher’s Hospital for Children’s NICU is a 39-bed level 4 regional outborn referral center in Philadelphia, Pennsylvania, with 200−250 annual admissions. Our unit provides the highest level of neonatal intensive care for those requiring advanced surgical services, therapeutic hypothermia, extracorporeal membrane oxygenation, and pediatric subspecialty services.

This quality improvement initiative was deemed exempt by the Drexel University College of Medicine institutional review board. The inclusion criteria were all neonates admitted to our NICU <34 weeks gestation. Exclusion criteria included infants ≥34 weeks and those with <7-day NICU stay. This initiative used the plan-do-study-act (PDSA) model for improvement to achieve our smart aim. We collected baseline data between January 2019 and May 2020; the first PDSA cycle occurred between June 2020 and October 2021, and the second PDSA cycle occurred between November 2021 and January 2023. Notably, the electronic medical record system transitioned during this quality improvement initiative in February 2021.

Our NICU clinician team includes neonatal attendings, neonatal nurse practitioners, neonatal-perinatal fellows, and pediatric residents. Our NICU has two dedicated NICU pharmacists, a nutritionist, occupational and physical therapists (OT/PT), and child life specialists. Every patient has a nutrition, PT, and OT consults upon admission. Speech therapy services were consulted as needed. All major subspecialties, including pediatric endocrinology and orthopedics, are available. Our institution does not perform advanced bone imaging such as dual-energy x-ray absorptiometry or dedicated bone ultrasounds. Radiographs are obtained as clinically indicated, and if fractures are noted, a skeletal survey may be performed at the physician’s discretion, and orthopedics is consulted if indicated.

### Interventions

**Fig. 1.**
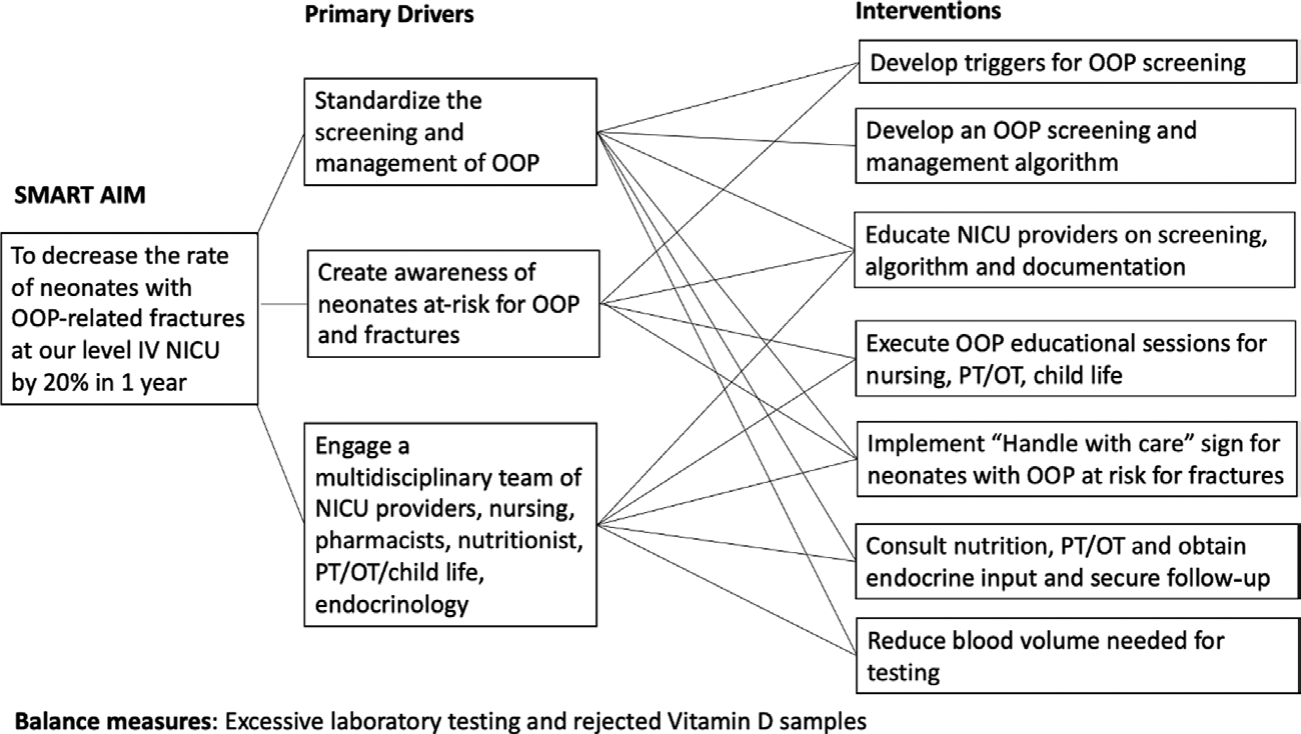
Key driver diagram. Key driver diagram depicting primary drivers and interventions to decrease the rate of neonates with OOP-related fractures at our level IV neonatal intensive care unit.

**Fig. 2.**
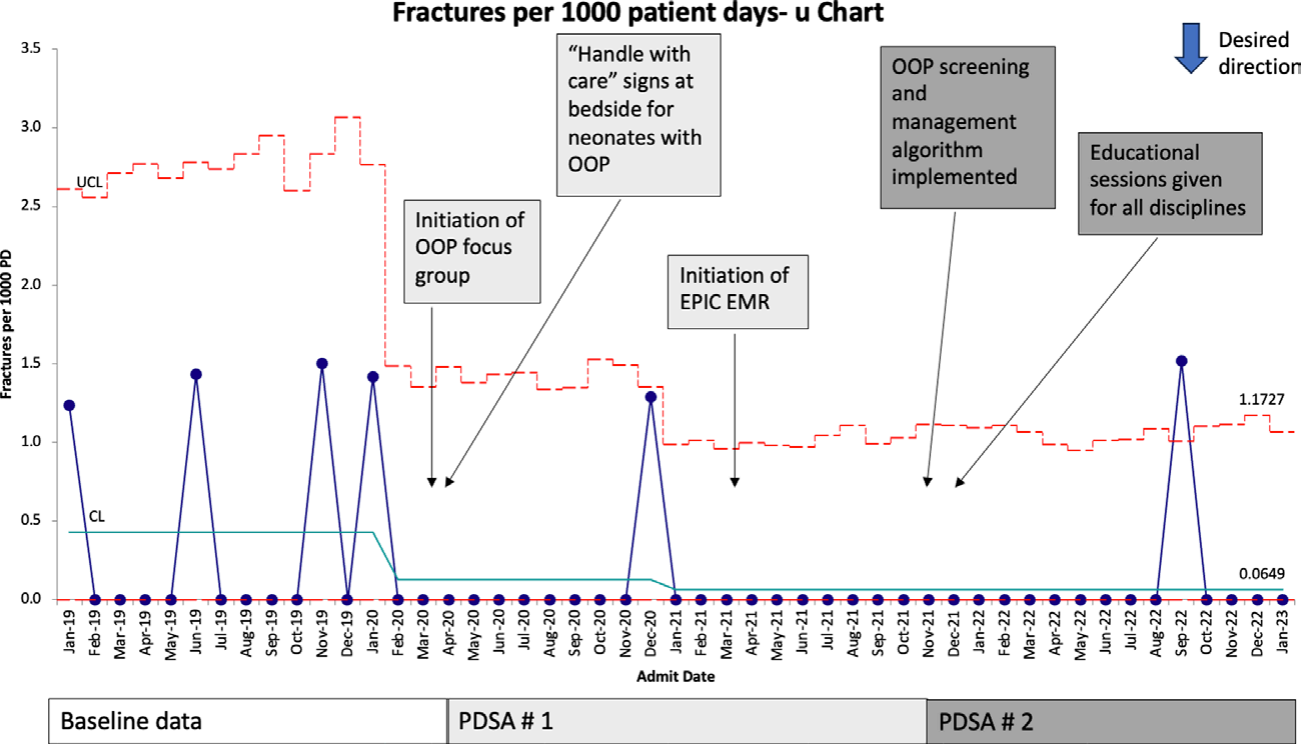
Outcome measure: rate of fractures. U chart demonstrating the reduction in the total number of fractures per 1000 PD after 2 PDSA cycles.

A multidisciplinary quality improvement team involving neonatologists, a neonatology fellow, an endocrinologist, NICU nursing educators, nurses, pharmacists, a dietician, physical therapists, occupational therapists, and a child life specialist was assembled in March 2020. A pareto analysis identified history of birth weight less than 1500 g, demineralization medication use, prolonged PN, lack of OOP screening, and delayed diagnosis as primary drivers for fractures (**See figure 1, Supplemental Digital Content**, which shows a pareto chart depicting the primary drivers for fractures include prescription of demineralizing medications, birth weight less than 1500g, and lack of OOP documentation. Other contributors included prolonged TPN, lack of OOP screening at 4-6 weeks and missed/delayed diagnosis. http://links.lww.com/PQ9/A545). A focus group held in May 2020 also revealed barriers to OOP screening, which included acute illnesses taking precedence over OOP screening, lack of documentation, unclear definitions, and tests requiring large blood volumes. A fishbone diagram was created (**See figure 2, Supplemental Digital Content 2**, which shows a fishbone diagram depicting the potential causes for osteopenia of prematurity related fractures. http://links.lww.com/PQ9/A545). With this information, a driver diagram was designed to identify key drivers and interventions to reduce OOP-related fractures (Fig. 1).

#### OOP Screening, Diagnostic, and Management Algorithm

**Fig. 3.**
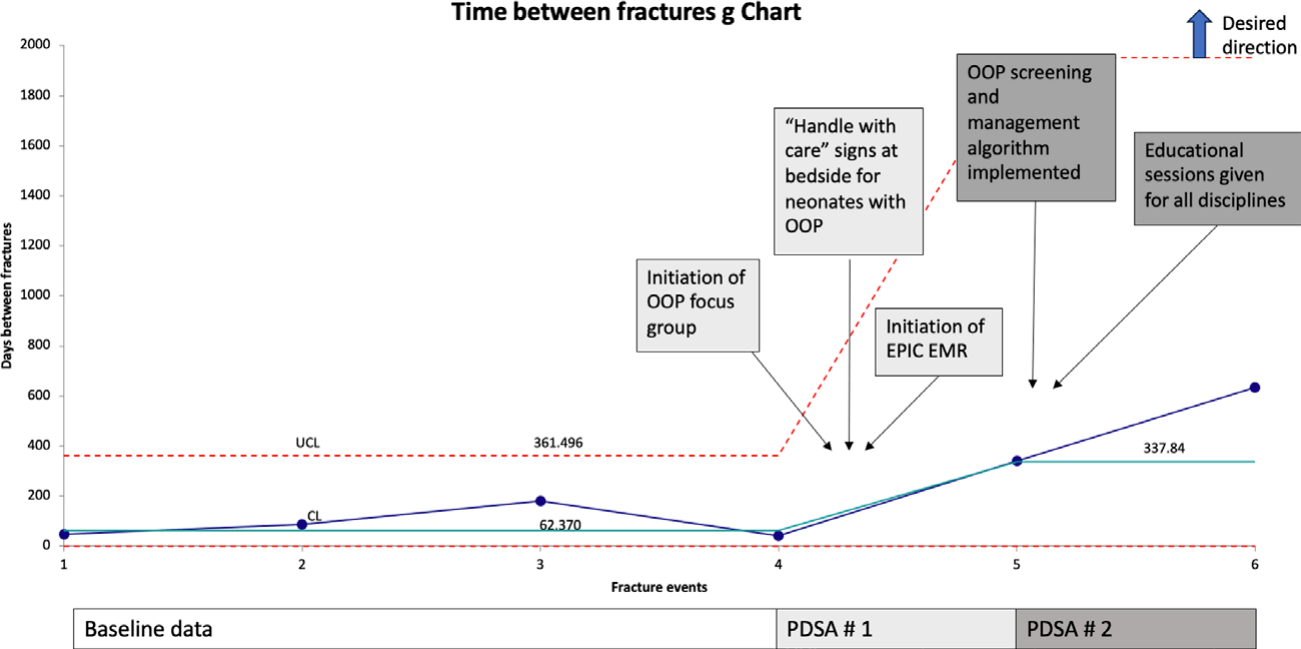
Outcome measure: time between fractures. G chart demonstrating an increase in the days between fractures after interventions.

We created the OOP screening and management algorithm after an extensive literature review in conjunction with neonatology, pediatric endocrinology, nutrition, and pharmacy (**See figure 3, Supplemental Digital Content 3**, which shows our local screening and management algorithm including patient at risk, timing of osteopenia screening, follow-up and management interventions. http://links.lww.com/PQ9/A545). The algorithm highlights infants at risk for OOP, recommended screening methods, timeframe and frequency of laboratory testing, guidance on management based on laboratory results, including nutritional interventions and recommended mineral supplementation.

For this initiative, an infant is defined as at risk for OOP if born <34 weeks gestation. We defined OOP as two consecutive laboratory values with an ALP >500 IU/L with or without a phosphate level <5.5 mg/dL and calcium level <8.5 mg/dL 1 week apart.^4,10^. If an infant has an ALP >500 IU/L, laboratory testing is repeated in 1 week, and if ALP >500 IU/L persists despite nutritional interventions, the infant is diagnosed with OOP. The diagnosis will be added to the problem list, and second-tier testing will be obtained within 24 hours as per the algorithm. For complex cases or those failing to improve per management algorithm, an endocrinology consult is recommended, and appropriate outpatient follow-up is secured as needed for discharge.

#### Laboratory Blood Volume

A common barrier noted by clinicians and nurses for OOP testing was the large blood volumes required, especially for Vitamin D levels. Historically, at least 1 ml of blood and a central stick were required for such testing. The OOP team discussed this with the laboratory. In January 2022, education was disseminated to reduce the needed blood sample to 0.6 ml, which could be obtained via heel stick.

#### Education

We conducted educational sessions to create awareness about OOP, fractures in our NICU, evidence-based medicine topic review, and the newly proposed screening and management algorithm. An educational poster was displayed in the NICU and PT/OT departments. Sessions were conducted by nursing, the project lead NICU fellow, and the champion endocrinologist at various times from November 2021 to November 2022.

#### “Handle with Care” Bundle

**Fig. 4.**
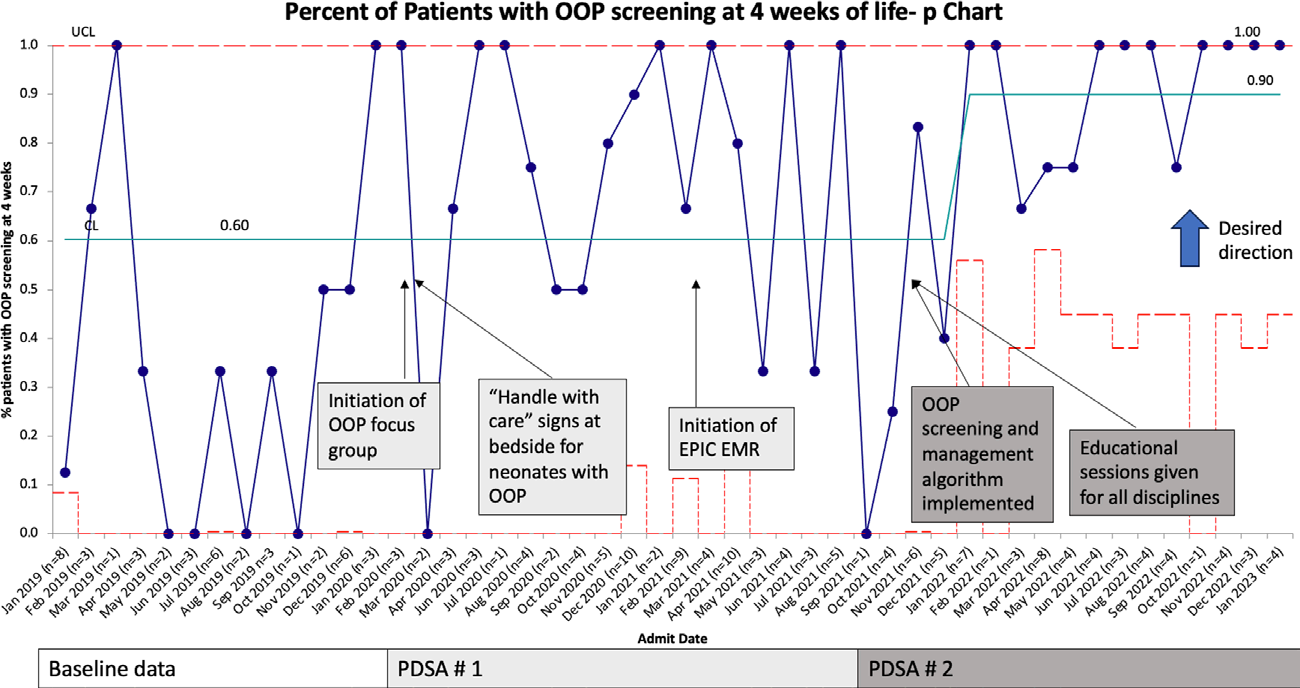
Process measures: OOP screening. Statistical process control chart demonstrating improvement in OOP screening laboratories obtained at four weeks of life after Interventions.

We created a “Handle with care” sign to be placed at infant’s bedside once an infant met the criteria for OOP to highlight the risk for fractures and that handling with care is needed for procedures and routine care. Multiple iterations of the sign were trialed with the final version, including the patient’s label at the top right corner and placed at eye level at the bedside to improve visibility (**See figure 4, Supplemental Digital Content 4**, which shows “handle with care” sign placed at infant’s bedside once they met criteria for OOP to highlight risk for fractures during routine care and procedures. http://links.lww.com/PQ9/A545). At the time of OOP diagnosis, clinicians updated the PT/OT electronic medical record consult to include the diagnosis of OOP to ensure mobility exercises and handling with care are performed.

### Study of the Interventions

Outcome measures included OOP-related fractures per 1000 patient days (PD), days between fractures, and the time to normalization of ALP. Process measures included patients between missed OOP documentation, percentage of algorithm compliance (defined as obtaining OOP screening laboratories at four weeks of age), and compliance with “handle-with-care” sign.

Balance measures included the monthly percentage of excessive screening laboratory testing and rejected Vitamin D samples for insufficient quantity. Excessive laboratory tests were defined as laboratory tests obtained more frequently than every other week in at-risk infants and greater than weekly in those with a diagnosis of OOP.

Data collected included neonatal demographics and characteristics, including comorbidities such as bronchopulmonary dysplasia, necrotizing enterocolitis, prolonged medication use (ie, diuretic, corticosteroid, and caffeine), and prolonged PN. Prolonged medication use was defined as 14 days or greater of consecutive use or two or more courses totaling 14 days or greater. Prolonged PN use was defined as 14 or more consecutive days of PN.

#### Statistical Analysis

Statistical process control charts were used to display process, outcome, and balancing measures using QI Macros 2021. Standard rules for special cause were applied.^8,9^ Pre and postintervention data were compared using chi-square test for categorical data and student *t* test for nominal data using SPSS, version 29. A *P* value of <0.05 was considered statistically significant.

## RESULTS

A total of 194 infants were included in this initiative: 51 infants in the baseline period and 143 infants in the postintervention period. The prevalence of OOP was similar between the baseline (31.3%, 16/51) and postintervention (30.1%, 43/143) groups.

Infants with OOP in both groups had similar mean gestational weeks and birth weights (Table 1). The baseline group had higher use of prolonged PN (87.5% versus 39.5%, *P* = 0.001), and there was no difference in the diagnoses of NEC (31.2% versus 18.6%, *P* = 0.311).

**Table 1.** Neonatal Characteristics including Comorbidities of Patients with OOP in the Baseline Group and Postintervention Group

Characteristics of Infants with Osteopenia	Baseline (n = 16)	Postintervention (n = 43)	*P*
Gestational age (wk), mean	25.4 (±1.7)	26.1 (±2.5)	0.341
Birth weight (g), mean	725 (±204)	789 (±289)	0.347
Sex, n (%)	8 (50)	18 (69)	0.769
Bronchopulmonary dysplasia, n (%)	15 (93.8)	40 (93)	1.00
Necrotizing enterocolitis, n (%)	5 (31.2)	8 (18.6)	0.311
Prolonged steroid course, n (%)	8 (50)	19 (44.2)	0.773
Prolonged diuretic use, n (%)	7 (43.8)	29 (67.4)	0.135
Maintenance caffeine, n (%)	16 (100)	43 (100)	NA
Prolonged PN, n (%)	14 (87.5)	17 (39.5)	**0.001** *
Calcium supplementation, n (%)	7 (43.8)	16 (37.2)	0.766
Phosphorous supplementation, n (%)	5 (31.2)	8 (18.6)	0.311

* *P* < 0.05 is statistically significant.

### Outcome Measures

With our interventions, we saw special cause improvement with a reduction in the rate of fractures from 0.43 per 1000 PD to 0.06 per 1000 PD (Fig. 2). Days between fractures revealed special cause improvement with a center-line shift from 62 to 337 days (Fig. 3). In the postintervention phase, we had two infants with fractures. Both were admitted with a diagnosis of OOP and had elevated ALP levels for over 30 days before admission.

We studied time to ALP normalization to measure algorithm effectiveness indirectly. While the proportion of patients with time to ALP normalization <16 days increased from 14.3% to 36%, normalization between 16 and 30 days decreased from 35% to 16%, and normalization >30 days remained unchanged (50% versus 48%) postintervention. Such differences were not statistically significant (*P* = 0.229).

### Process Measures

**Fig. 5.**
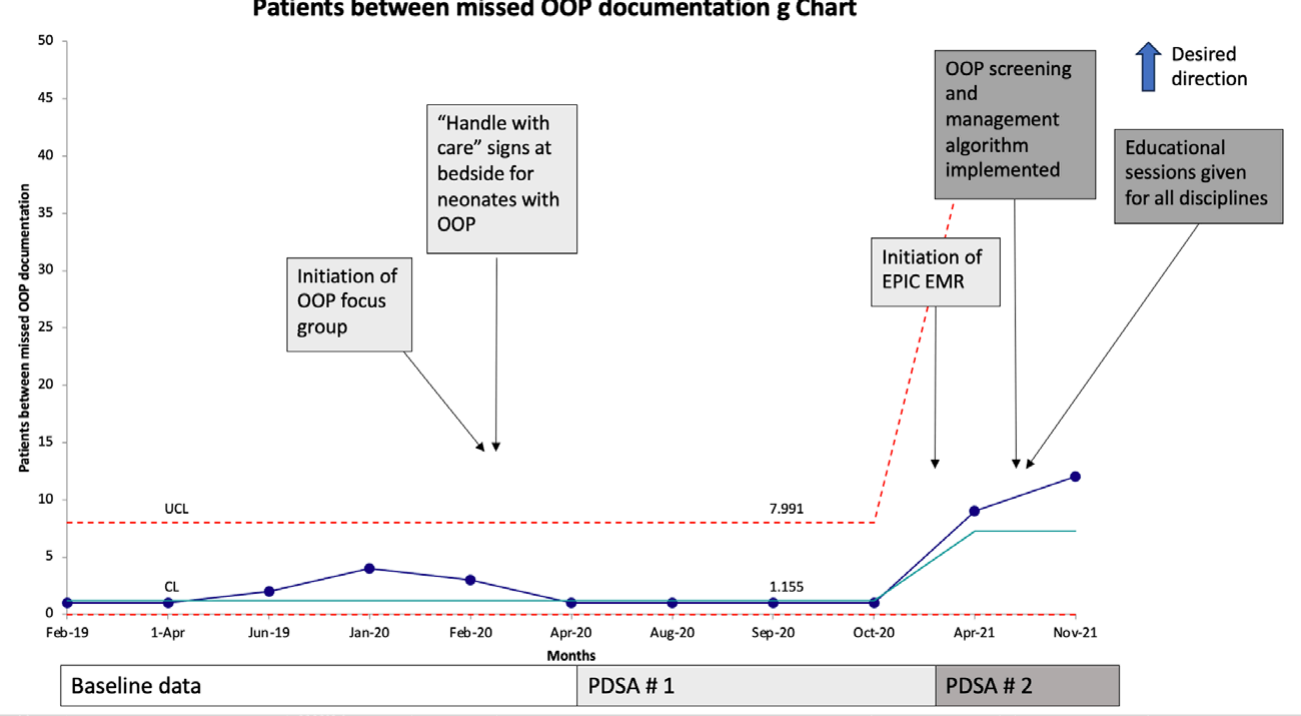
Process measure: documentation of OOP diagnosis. G chart demonstrating improvement in patients between OOP diagnosis documentation with interventions.

There was special cause variation in the percent of screening OOP laboratories obtained at four weeks of age, which increased from 60% to 90% after 2 PDSA cycles (Fig. 4). We saw special cause improvement in the number of patients between missed OOP documentation with a center-line shift from 1.2 to 7.3 after interventions (Fig. 5). Compliance with the handle-with-care signs at the bedside of patients with OOP and PT/OT consults was 100%.

### Balancing Measures

The percentage of patients undergoing excessive testing remained unchanged throughout the initiative at 16%. No Vitamin D samples were deemed as quantity insufficient after implementing the 0.6-ml blood volume requirement.

## DISCUSSION

A multidisciplinary team approach standardizing the screening, diagnosis, and management of neonates at risk of/with OOP combined with a handle-with-care bundle led to the reduction of fractures in our referral level IV NICU. We achieved this improvement despite a similar prevalence of OOP diagnosis throughout this initiative and without excessive testing.

Although fractures are a rare event, they can lead to increased morbidity and impact long-term growth. Many of the guidelines available focus on OOP screening and prevention. A challenge for referral NICUs, such as ours, relates to the admission of infants at varied postnatal ages, often beyond the preventative stage of many diagnoses, including OOP. Therefore, our multidisciplinary team focused on OOP screening and management to provide necessary interventions in a timely fashion to reduce fractures.

Serum ALP levels greater than or equal to 900 IU/L had 100% sensitivity and 70% specificity for MBDP.^3^ A more conservative ALP cutoff of >500 IU/L has been proposed as a cutoff for the risk of developing MBDP.^4^ While calcium is an important mineral for bone growth, these levels are not specific for determining bone demineralization in isolation.^4^ On the other hand, hypophosphatemia is a superior measure of bone demineralization and, in conjunction with calcium and ALP levels, can be used to determine if the infant has OOP.^4^ Additional tests such as parathyroid hormone, tubular reabsorption of phosphorous, and vitamin D levels can help guide management, specifically supplementation.^3,4^ These tests often require higher blood volumes, which may limit their practicality in caring for premature neonates.

Other institutions have similarly embarked on efforts to reduce OOP fractures. Mohamed et al created a nutritional monitoring and interventional protocol for OOP and observed a decrease in peak ALP levels and a decreased fracture rate by establishing guidelines for calcium and phosphorous supplementation.^10^ In 2019, Faienza et al proposed general screening and management guidelines for OOP based on the evaluation of individual biomarkers.^2^ However, their guideline did not indicate laboratory frequency or follow-up. Our NICU baseline data also revealed screening and frequency of laboratory monitoring varied greatly; thus, such guidance became a priority in the multidisciplinary development of our algorithm.

One of the significant differences in our diagnostic criteria compared with other studies is the ALP level. While levels greater than 500 IU/L have indicated disruption in bone homeostasis, levels greater than 800 IU/L are associated with radiographic findings of bone demineralization.^11^ Viswanathan et al demonstrated levels greater than 500 IU/L in extremely low birthweight infants less than 30 weeks’ gestation was associated with OOP.^12^ When analyzing our baseline group, several infants had multiple risk factors for OOP, such as prolonged diuretic use, corticosteroid use, and PN. Thus, given the complexity of our cohort, we chose a lower ALP threshold to capture infants most at risk and intervene before the development of additional morbidities.

We noted a shift in the time to ALP normalization during this initiative, albeit not statistically significant, indirectly suggesting our screening and management guideline possibly mitigated prolonged ALP elevation in a subgroup of patients. The proportion of patients normalizing ALP between 16 and 30 days decreased while the proportion of normalizing before 16 days increased. This may represent a group of patients with transiently abnormal ALP that respond to nutritional interventions as suggested by the algorithm. We posit the lack of change in the >30-day group may represent the broad range in days to ALP normalization, patients with persistently elevated ALP to the time of discharge and those on prolonged demineralizing medications/PN. Targeted interventions in this group of patients may help further reduce fractures in our NICU, and continued surveillance may aid in detecting statistical significance in the time to ALP normalization.

Another significant difference in our guideline was immediate vitamin D supplementation once infants were receiving enteral feeds. Applebaum et al implemented a bone health screening protocol that monitored only ALP, vitamin D levels, and wrist radiographs with notable improvement in OOP diagnosis from 50% to 100%.^13^ Studies have varied on whether vitamin D supplementation alone prevents OOP but have agreed that calcium and phosphorous supplementation have a more significant impact. While Vitamin D levels in our initiative were obtained as part of secondary tier laboratories, it is important to note that Vitamin D supplementation did not have to be adjusted in any of our patients. Additionally, we obtained this laboratory value using less blood volume than previously, which validated clinician concerns and engaged them as integral initiative members. Given Vitamin D levels did not tangibly change the management for our particular patient population, streamlining second-tier laboratory testing to exclude Vitamin D levels may be an area for future consideration. In the cohort studied, the prevalence of OOP diagnosis did not change despite early vitamin D supplementation and our interventions. Such a finding underscores the need for additional nutritional strategies and minimization of bone demineralizing medications to better address OOP prevention strategies among such critically ill patient population.

The second largest intervention that led to the success of this initiative was the “handle with care” bundle. Baseline data revealed infants with fractures also had recent procedures, such as central line placement in the same extremity as the fracture. In addition, immobilization is a known risk factor for OOP, and specific exercises can be used to promote bone mineralization.^14^ “Handle with care” signs were placed at the infant’s bedside if they met the criteria for OOP to serve as a reminder that these infants were at high risk for fractures. Multidisciplinary involvement of PT, OT, child life, and nurses was crucial in ensuring 100% compliance with the signs and mobility exercises.

Another strength of this initiative was the availability of subspecialty services in a level IV NICU. Our institution has the benefit of easy access to in-house pediatric endocrinology, a dedicated nutritionist, and a pharmacist in the NICU for input during daily rounds as well as PT, OT, and child life services. The intricate collaboration amongst all these disciplines allowed for unit-wide involvement, improved compliance with our interventions, and a reduction in fractures in this high-risk population.

### Limitations

This quality improvement initiative was conducted in a level IV NICU; therefore, the resources and support available in our unit may not be present in all NICUs, possibly limiting its generalizability. However, the OOP screening and management guideline and “handle with care” sign are easily reproducible interventions that can apply to NICUs caring for the preterm population. Our initiative utilized two ALP levels >500 IU/L as diagnostic for OOP based on our baseline data to include our at-risk for OOP-related fractures without radiologic confirmation. While the gold standard for bone demineralization diagnosis is dual-energy x-ray absorptiometry, such specialized imaging is not readily available, such as in our institution.^15^ While x-rays are often obtained in NICU patients, comments on bone mineralization were often not included in our patients and, therefore not captured in this initiative.

Next steps include focusing on nutritional strategies and reducing the use of bone demineralization medications to aid in the prevention of OOP. Krithika et al focused on providing early calcium and phosphorous supplementation through PN and fortification, and with this, they observed a reduction in OOP from 35% to 17%.^16^ Therefore, becoming more intentional in calcium and phosphorous supplementation in PN and enteral nutrition may aid in the continued sustainability of our efforts in reducing fractures and overall prevention of OOP.

## CONCLUSIONS

A multidisciplinary team approach with standardized OOP screening and a management guideline that includes a “handle with care” bundle reduces OOP-related fractures in a level IV NICU. The next steps include nutritional strategies to optimize calcium and phosphorous delivery and reduction of demineralizing medication use to aid in preventing OOP.

## ACKNOWLEDGMENTS

The authors acknowledge the following personnel for assistance with the study: neonatal attendings, fellows, nurse practitioners and nurses, as well as the physical and occupational therapists and child life at St. Christopher’s Hospital for Children.

## Supplementary Material


