# Supplementary material for: Reducing Osteopenia of Prematurity-related Fractures in a Level IV NICU: A Quality Improvement Initiative
Source: Pediatr Qual Saf. 2024 Apr 3;9(2):e723. doi: 10.1097/pq9.0000000000000723 (PMC10990331; doi:10.1097/pq9.0000000000000723)
Supplement: Supplementary file 1 [file pqs-9-e723-s001.pdf]

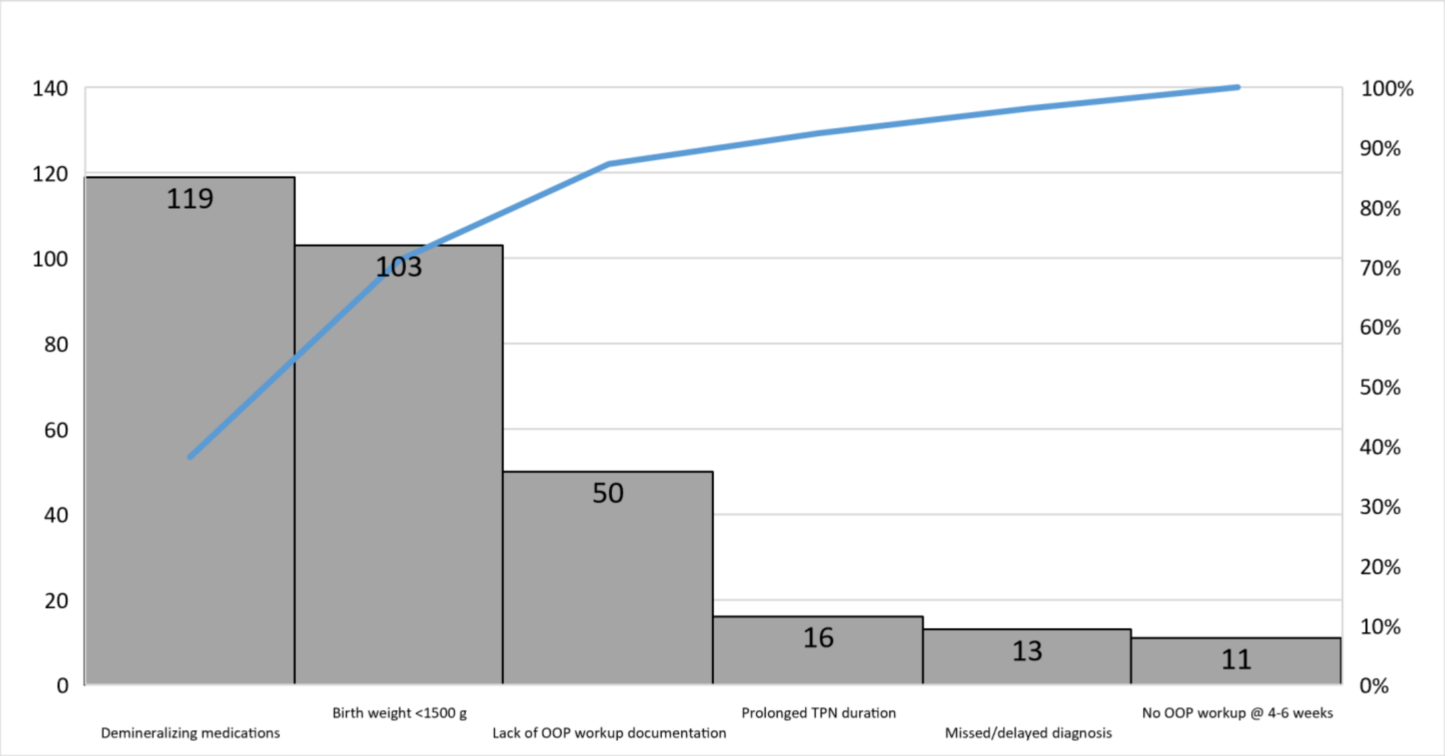

Supplemental Figure 1.
A pareto chart depicting the primary drivers for fractures include prescription of demineralizing medications, birth weight less than 1500g, and lack of OOP documentation. Other contributors included prolonged TPN, lack of OOP screening at 4-6 weeks and missed/delayed diagnosis.

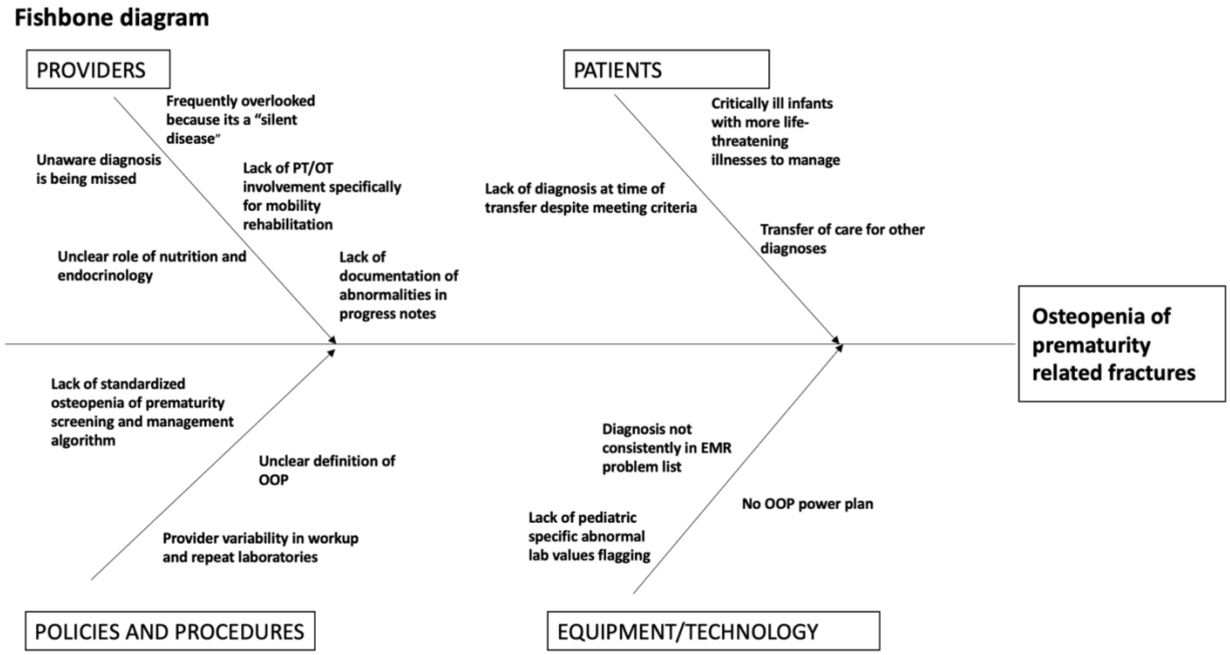

Supplemental Figure 2.
A fishbone diagram depicting the potential causes for osteopenia of prematurity related fractures.

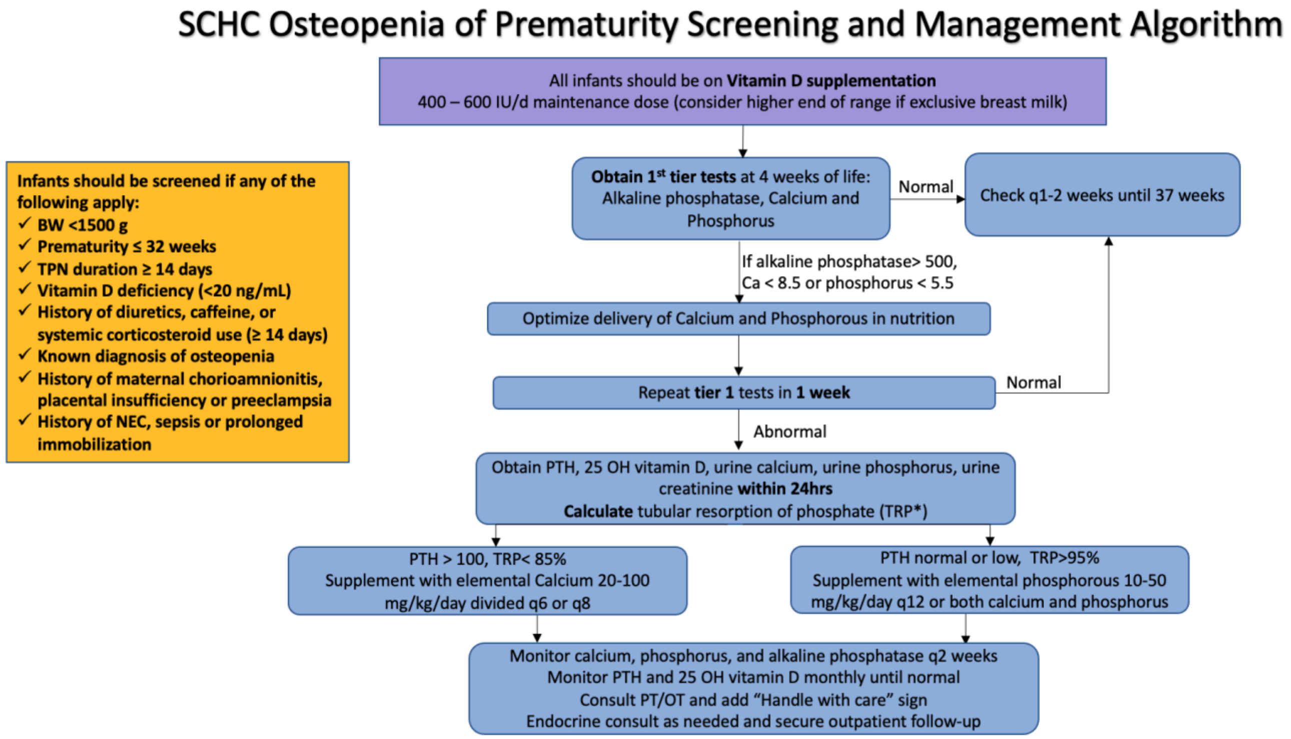

* TRP (tubular resorption of phosphate)=[1 - (urinary phosphorus/urinary creatinine × serum creatinine/serum phosphorus)] × 100

Supplemental Figure 3.
Our local screening and management algorithm including patient at risk, timing of osteopenia screening, follow-up and management interventions.

Please

HANDLE ME WITH

Extra Gentle Care

I MAY HAVE FRAGILE BONES

Care and Procedures

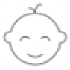

- Handle me with slow, gentle movements.
- Do not pull, push or twist my limbs, especially with IV/line insertion or blood draws.
- Avoid applying pressure during axillary temperatures.

Lifting

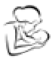

- When lifting or repositioning me, use one hand to support my head and trunk and the other hand to support under my buttocks.

Diapering

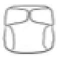

- Avoid lifting me by my ankles when changing my diaper. Instead, slide your hand under by buttocks to lift me to remove/replace my diaper, or change my diaper in a side lying position.

Dressing

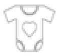

- Please dress me in loose clothing.
- When dressing and undressing, gently pull the clothing over my arms/legs instead of pulling my arms/legs through the clothing.

Swaddling

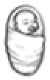

- When swaddling me with a blanket or sleep sack, avoid bundling too tightly since this could cause pressure on my arms/legs.

Feeding and Burping

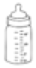

- When burping me, pat me gently using soft taps with a cupped hand.

Supplemental Figure 4.

Handle with care" sign placed at infant's bedside once they met criteria for OOP to highlight risk for fractures during routine care and procedures.
